# Measuring the Flight Trajectory of a Free-Flying Moth on the Basis of Noise-Reduced 3D Point Cloud Time Series Data

**DOI:** 10.3390/insects15060373

**Published:** 2024-05-21

**Authors:** Koji Nishisue, Ryo Sugiura, Ryo Nakano, Kazuki Shibuya, Shinji Fukuda

**Affiliations:** 1 Institute of Agriculture, Tokyo University of Agriculture and Technology (TUAT), 3-5-8 Saiwai-cho, Fuchu-shi 183-8509, Tokyo, Japan; nishisue.koji@gmail.com; 2 The Research Center for Agricultural Information Technology (RCAIT), National Agriculture and Food Research Organization (NARO), 2-1-9 Kannondai, Tsukuba-shi 305-0856, Ibaraki, Japan; rsugiura@naro.affrc.go.jp; 3 Institute for Plant Protection (NIPP), National Agriculture and Food Research Organization (NARO), 2-1-18 Kannondai, Tsukuba-shi 305-8666, Ibaraki, Japan; rnakano@naro.affrc.go.jp (R.N.); shibuyak592@naro.affrc.go.jp (K.S.)

**Keywords:** 3D point cloud, flight trajectory, free flight, pest control, *Spodoptera litura*, stereo vision

## Abstract

**Simple Summary:**

Pest control plays an important role in crop production. The cotton leafworm, *Spodoptera litura*, is well recognized as a pest that causes severe damage to a wide variety of crops. Because *S. litura* is nocturnal, it is challenging to control this species effectively. Recently, laser zapping has gained attention as a clean technology to control pest insects. It is important to precisely identify and predict the flight trajectories of free-flying moths under low-light conditions for better sighting during laser zapping. In this study, we developed an automatic detection pipeline based on point cloud time series data from stereoscopic images. Three-dimensional point cloud data were extracted from disparity images recorded under infrared and low-light conditions. We computed the size of the outline box and the directional angle of the 3D point cloud time series to remove noisy point clouds. We visually inspected the flight trajectories and found that the size and direction of the outline box were good indicators of the noisy data. Finally, we obtained 68 flight trajectories, and the average flight speed of free-flying *S. litura* was found to be 1.81 m/s.

**Abstract:**

Pest control is crucial in crop production; however, the use of chemical pesticides, the primary method of pest control, poses environmental issues and leads to insecticide resistance in pests. To overcome these issues, laser zapping has been studied as a clean pest control technology against the nocturnal cotton leafworm, *Spodoptera litura*, which has high fecundity and causes severe damage to various crops. For better sighting during laser zapping, it is important to measure the coordinates and speed of moths under low-light conditions. To achieve this, we developed an automatic detection pipeline based on point cloud time series data from stereoscopic images. We obtained 3D point cloud data from disparity images recorded under infrared and low-light conditions. To identify *S. litura*, we removed noise from the data using multiple filters and a support vector machine. We then computed the size of the outline box and directional angle of the 3D point cloud time series to determine the noisy point clouds. We visually inspected the flight trajectories and found that the size of the outline box and the movement direction were good indicators of noisy data. After removing noisy data, we obtained 68 flight trajectories, and the average flight speed of free-flying *S. litura* was 1.81 m/s.

## 1. Introduction

Damage caused by pests is one of the main causes of loss in agricultural production [1]. Feeding damage caused by lepidopteran insects is particularly significant. *Spodoptera litura* (Fabricius) (Lepidoptera: Noctuidae) is a major pest in the Asia–Pacific region [2]. It is a euryphagous insect that feeds on a wide variety of crops [3], it has a high reproductive rate, and some populations have insecticide resistance [3,4], with middle- and older-instar larvae having particularly low susceptibility. At present, the management of *S. litura* is generally carried out by chemical control using insecticides. However, it is difficult to effectively control *S. litura* by chemical control alone, such as through the spraying of agricultural chemicals, because of its resistance to insecticides and the ecology that *S. litura* is generally nocturnal, and the last instar larvae and pupae hide in the soil [5,6]. Therefore, integrated pest management (IPM), which does not rely solely on chemicals, is required to control *S. litura*. Delta endotoxins (Bt toxins) and nuclear polyhedrosis virus (NPV) have shown some success as a biological control [7,8]; however, the practical use of physical control is limited to primary methods such as blocking with insect nets.

In recent years, laser zapping has been studied and proposed as a new physical pest control method [9,10,11,12,13,14,15,16,17]. Laser zapping has also been studied for controlling *S. litura* [18,19]. For efficient laser zapping, it is necessary to accurately measure flight trajectories and speed and to predict the trajectories a few steps ahead. In addition, because this species is generally nocturnal, its flight trajectories cannot be observed and recorded with a camera using visible light. Therefore, it is necessary to develop a system that can measure flight trajectories and speed under dark or low-light conditions. In addition, basic ecological information, such as flight trajectory and speed, is important for controlling flying insect pests. For nocturnal flying insects such as *S. litura*, observation methods are particularly limited, and new methods need to be developed. Such measurement technology enables the investigation and provision of ecological information, not only in applied science, such as pest control, but also in basic science.

Therefore, this study aims to measure the flight trajectories and speed under low-light conditions by recording videos of the flight of *S. litura* using infrared light and a stereo camera and converting them into three-dimensional (3D) point cloud time series data. In this study, all of the “point clouds” were generated from disparity images. Because the 3D point cloud data obtained from these disparity images contained numerous noise data, we removed them by multistep processing by considering background noise, the size of the point cloud, and the length of the point cloud time series and using a noise classifier with flight trajectory. The remaining noise was removed from the selected flight trajectories by using 3D animation and descriptive statistics of the 3D point cloud time series. We considered the flight speed from the noise-free flight trajectory data of free-flying *S. litura* as their flight speed and compared it with data from previous studies.

## 2. Materials and Methods

### 2.1. Sample Collection and Rearing

We fed an artificial diet (Insecta LFM, Nosan Co., Yokohama, Japan) to *S. litura* larvae collected from sweet potatoes and reared them in Tsukuba City, Ibaraki Prefecture, Japan. The larvae were maintained at 24 °C. We used virgin females 2–4 days after eclosion in all flight experiments.

### 2.2. Video Recording Using Stereo Camera

**Figure 1:**
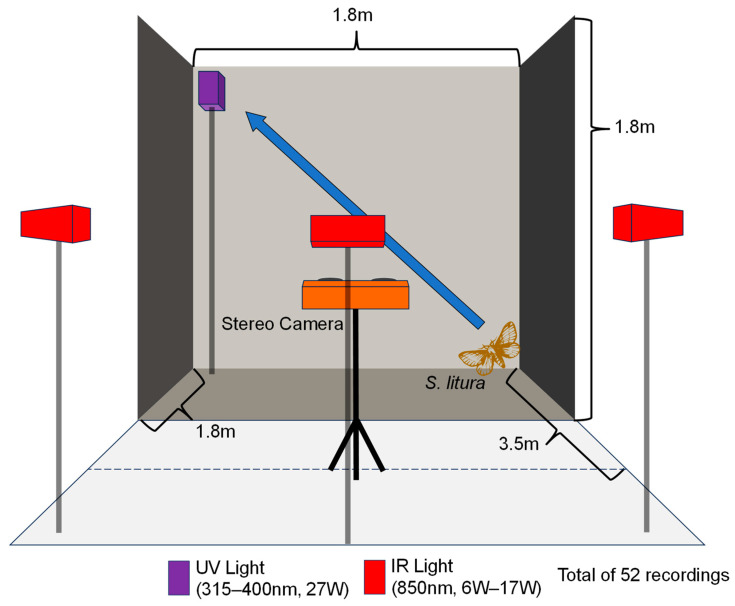
A schematic diagram of the video recording environment. A view of the recording arena from the side of stereo camera. The height of the arena was set at 1.8 m. The recording arena was maintained at 24 °C in an indoor laboratory. During the recording, only infrared and ultraviolet lights were turned on for recording and stimulating light resources, respectively. Visible light was turned off and blocked.

We set up the recording video space to be 1.8 m high × 1.8 m wide × 3.5 m deep (Figure 1). The recording space was maintained at 24 °C. It was enclosed in a mosquito net to prevent escape from the laboratory room, and the insects only flew in the recording area. The net was a dark black color, not lustrous, and not completely smooth. It was visually displayed as a wall from a visible camera. A stereo camera (Table 1, Karmin3 with SceneScan Pro, Nerian Vision Technologies, Stuttgart, Germany, 55 fps) was installed at the center of the short side on one side. Three infrared lights (850 nm, 6 W, 10 W, and 17 W, unbranded) were installed on the same side as the light sources to record the stereo camera images. Ultraviolet light (315–400 nm, 27 W, FPL27BLB, SANKYO DENKI Co., Hiratsuka, Japan) was installed on the opposite side to stimulate free flight in *Spodoptera litura*. Red light was used as the working light source. We only turned on infrared light as a light source and ultraviolet light as a stimulating light during video recording, and we turned off or blocked visible light, including the working light.

The experiment was conducted under free-flight conditions, without using a flight mill or tethering with a thread. *S. litura* was released from a position in the center of the long side of the recording area, and recordings were performed for about 5 s until it flew out of the recording range (sufficient to observe a flight time of about 3 s at the maximum). We obtained 52 flights recorded under these conditions. Of the 52 recordings, one individual was released per recording in 48 of them, and multiple individuals were released simultaneously in 4 of them (three individuals twice, four individuals once, and seven individuals once). After confirming that the data from multiple releases could be handled in the same way as the data from a single release, the data were processed using the same methods for subsequent analyses.

### 2.3. Extracting Spodoptera litura Flight Trajectories

**Figure 2:**
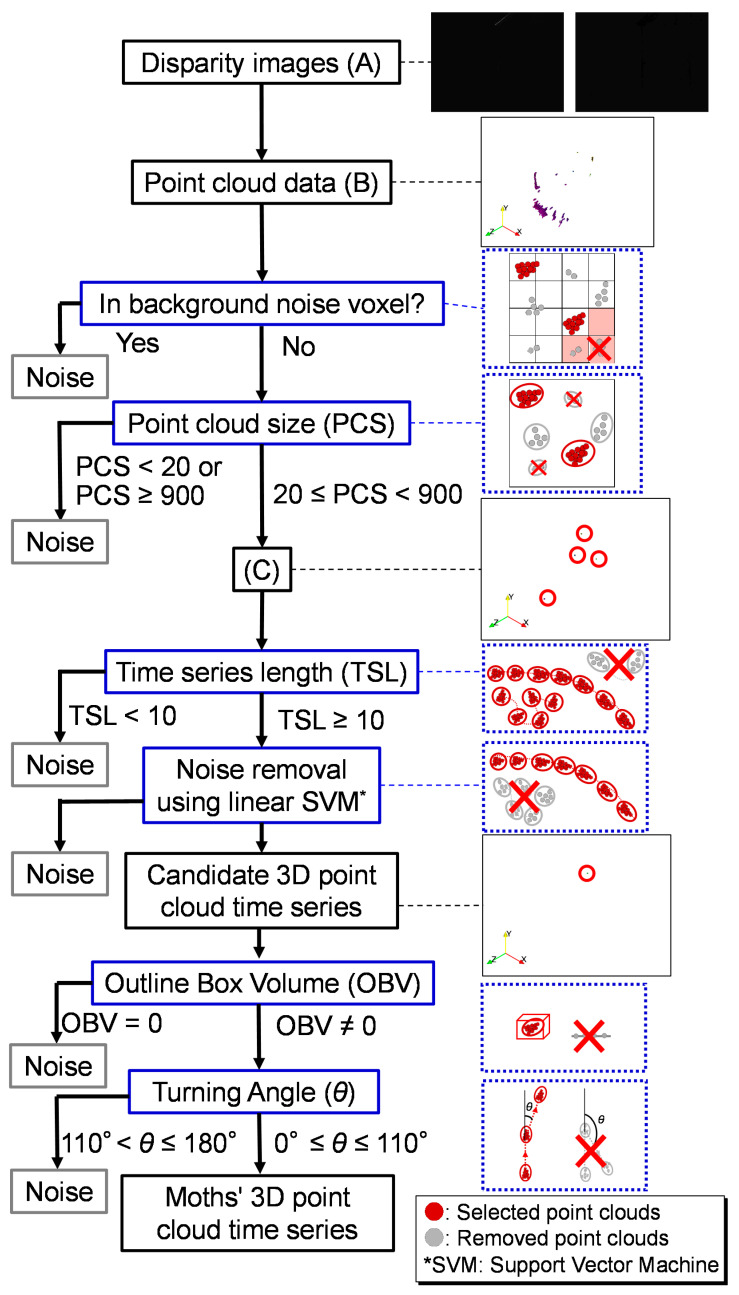
A schematic diagram of the noise removal pipeline process. The series of images in (**A**–**C**) of the right column is an example of the corresponding data. The point cloud data obtained from the disparity images (**A**) contained a large number of noise point clouds (**B**). The pipeline process for selecting only *Spodoptera litura* point clouds by noise removal was performed as follows: In the first step, to remove the noise point clouds that were always displayed, the point clouds in the background noise voxels where point clouds constantly exist were removed. The background noise voxels consisting of a collectivity of boxes (2.5 cm units per side) that existed for the first four frames of recording were considered as noise and removed. In the second step, point clouds within 1 cm of the remaining point clouds were considered the same object, and point clouds with a point cloud size (PCS) of less than 20 or more than 900 were removed as noise (**C**). In the third step, point clouds spanning consecutive frames in the time series were considered the same object, and point clouds with a time series length (TSL) of less than 10 frames were considered noise and removed. In the fourth step, a linear support vector machine (SVM) based on the time series coordinate data was used for classification, and the remaining data were selected as candidate point clouds for *S. litura*. In the fifth step, point clouds with an outline box volume (OBV) of 0 were removed as abnormal data. Finally, point clouds with a turning angle greater than 110° were removed, and the remaining data were selected as *S. litura* point clouds. The red circles indicate the locations of the point clouds selected at each step. (**A**) A pair of disparity images of the original data recorded using a stereo camera. Because the stereo camera is not an infrared camera, the disparity images are almost exclusively visually black. However, point clouds can be obtained from the black images using this pipeline. (**B**,**C**) show images of point cloud data visualized using ParaView.

The 3D flight trajectory data were obtained using the following automatic detection pipeline [18] (Figure 2). The disparity images captured by the stereo camera were converted into 3D point cloud data, which were sequenced in time series order according to the following steps and criteria of the pipeline. The initial step in the filtering method involved identifying stationary objects as the background to remove the point cloud of background noise from these files. By utilizing cubic voxels with a size of 5 cm on each side and examining the first four frames, voxels containing points for at least two frames were designated as the background. All of the points within the background noise voxels were removed in the subsequent frames. The remaining points were grouped into the same clusters if the closest point was within 1 cm of each other, which could be considered an individual object. The second step of the pipeline eliminated clusters with fewer than 20 or more than 900 points, as they are unlikely to be moths from the size information of *S. litura*. The remaining objects were of similar size to the moths but could not be definitively identified as moths at this stage. In each frame, the mean coordinates of the points within a group were determined as the position of the object. The object closest to the object of interest was selected from the extracted objects in the following frame; if the distance between them was within 9 cm, the selected object was considered the object from the same real object. In addition to simple calculations from the ecological and/or morphological data of *S. litura*, the values of the points and distances were determined through trial and error. This process allows for the motion of an object to be captured over time as time series data. A linear support vector machine (SVM) was employed as a moth/noise classifier utilizing two input variables: the standard deviation of the time series positions of the object within the last 10 frames and the standard deviation of the angular variation of the vector between consecutive points. By computing some disparity images, the total processing time for this task was estimated to be approximately 22.2 ms, from capturing the disparity image to making a position prediction. To accurately target an object with a laser, it is necessary to compensate for this time lag. The stereo vision system updated the image at a rate of 55 fps, and one update required 18 ms. Therefore, two steps ahead (36 ms) of the prediction were employed in this system. The selected point cloud was treated as a candidate flight trajectory in the subsequent analysis.

### 2.4. Additional Noise Removal

#### 2.4.1. Three-Dimensional Animation of Three-Dimensional Point Cloud Time Series

The visualization software ParaView (Ver. 5.11.0) [20] was used for animating and visualizing the 3D point cloud time series as each of the flight trajectory candidates. Using these 3D animations, we visually checked the partial data to determine whether the point cloud data were *S. litura* data. If the data had continuous, smooth, and constant direction movements, we classified the time series data as appropriate for the true flight trajectory of *S. litura*. We manually removed the remaining discontinuous data and tended not to move the noise data. In addition, the 3D point cloud time series was converted from binary data (PLY files) to text data (CSV files) using ParaView. We used R (Ver 4.3.1) [21] on the text data to calculate the speed, turning angle, distance from the camera using centroids of measurement point cloud as well as the volume of the outline box containing the point cloud, the number of point clouds, and the point cloud density in each frame, of which descriptive statistics (i.e., mean, median, and quartiles) were calculated. The manual operations described in this section were performed to obtain information for improving the pipeline.

#### 2.4.2. Remaining Noise Removal Process

From the descriptive statistics of the 3D point cloud time series, we detected and removed the noise point cloud (Figure 2). The removal process is as follows: First, we selected point clouds with outliers in the outline box volume (OBV) of the 3D point cloud and/or outliers in the turning angle of the time series data in the interquartile range. Second, the time series point cloud with outliers was checked using 3D animation frame by frame, and the data including discontinuous data and/or few movement data were removed as noise data. This process was repeated from outside the distribution of the values until the noise data were no longer confirmed. Finally, the remaining data without noise were treated as *S. litura* trajectory point cloud data. The criteria of OBV and turning angle were determined using the information from the manual removal of noise processed in Section 2.4.1. In addition, we compared the statistical data of the *S. litura* point cloud data and the noisy point cloud data and examined the difference in features for improving the automatic flight trajectory processing pipeline.

## 3. Results

### 3.1. Three-Dimensional Point Cloud of Flight Trajectories

Most of the point clouds from the disparity images (Figure 2A) were noise point clouds, such as background noise (Figure 2B). A few point clouds were left in these files, in which constant background noise was removed (Figure 2C). These point clouds included *S. litura* point clouds that moved smoothly and continuously; noise point clouds were isolated and disappeared without moving. Most of the noise point clouds were removed in the final files of the pipeline. Although our program could obtain the true flight trajectory of *S. litura* from disparity images, it was found that the point clouds included not only the true flight trajectories, but also noisy point clouds, as a result of a visual inspection using 3D animation.

### 3.2. Removal of Remaining Noisy Point Cloud

**Figure 3:**
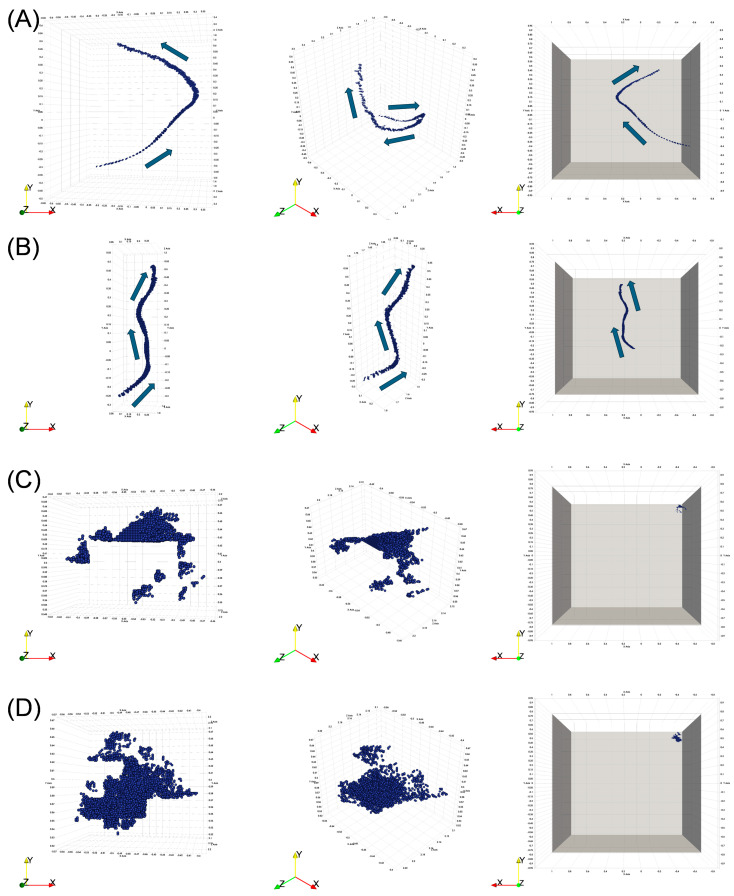
Examples of the entire three-dimensional (3D) point cloud time series in a dataset. The three columns show the same data for the different display angles. (**A**,**B**) are the *Spodoptera litura* flight trajectories, and (**C**,**D**) are the noise point clouds that remain after the pipeline. The frame sizes of each dataset are (**A**) 82 frames, (**B**) 65 frames, (**C**) 58 frames, and (**D**) 42 frames. The arrows in (**A**,**B**) indicate the direction of the movement based on the time series. There are no consecutive directions, but there are small distances of movement in (**C**,**D**). In the left and center columns, the scales are variable to fit the point cloud size. In the right columns, the scales are fixed, and the wall of the recording arena is added virtually. The units of the XYZ axis are m.

The 3D visualization of the flight trajectories indicated that the *S. litura* point clouds had continuous and smooth trajectories (Figure 3A,B), whereas the noise point clouds showed discontinuous and limited movement (Figure 3C,D). We found two parameters, namely the volume of the outline box and the turning angle based on time series, to be effective for noise detection. For instance, volume-less outline boxes (i.e., no thickness; volume = 0) were abnormal data in which all point clouds existed on the same surface. In addition, the turning angle of the 3D point cloud time series in the range of 110° < θ ≤ 180° contained noisy point clouds that repeated reciprocating motions in a fixed area.

**Figure 4:**
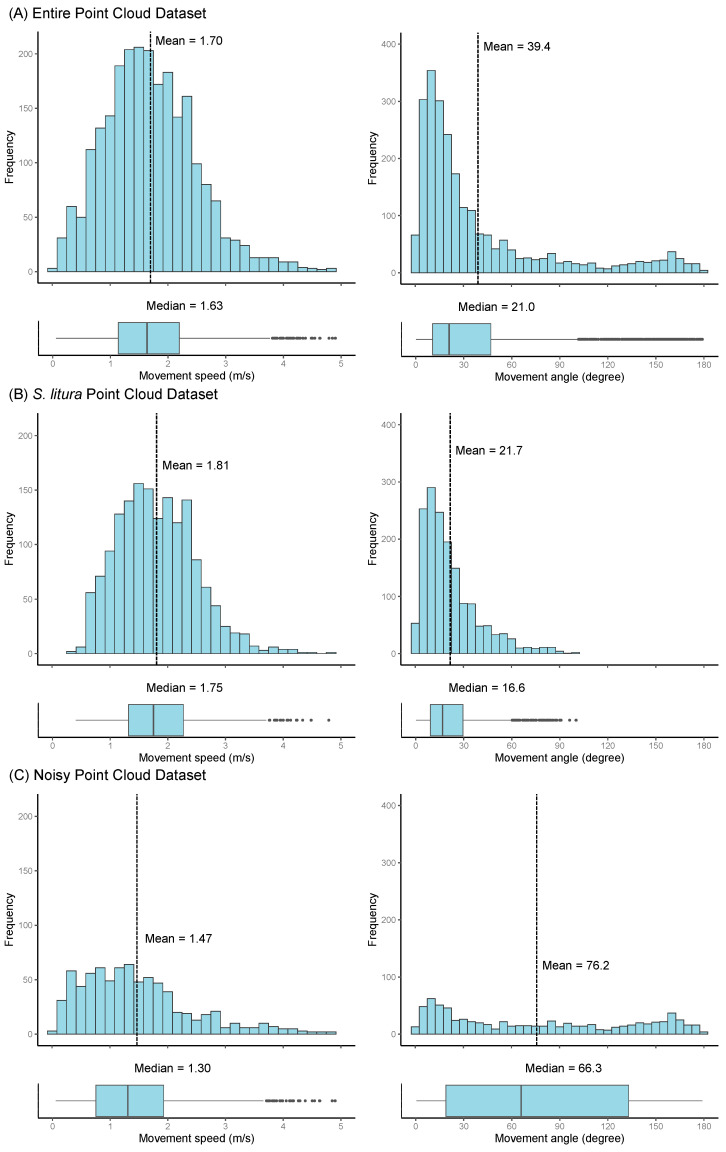
A histogram and boxplot of the flight speed and turning angle based on the centroids of the point cloud time series data: upper row (entire point cloud data) (**A**), middle row (*Spodoptera litura* point cloud data) (**B**), and lower row (noisy point cloud data) (**C**). The left and right columns represent the flight speed and turning angle, respectively. The dotted line in the histogram indicates the mean value, and the solid line in the boxplot indicates the median value.

We classified the obtained point cloud data into three categories, namely “entire point cloud data” (all point cloud data obtained by pipeline processing), “*S. litura* point cloud data” (only flight trajectory point cloud data from which remaining noise point clouds were removed from entire point cloud data), and “noisy point cloud data” (point cloud data containing noise removed in *S. litura* point cloud data). The entire point cloud data consisted of the *S. litura* point cloud data and the noisy point cloud data. When these three datasets are compared in terms of speed and turning angle (Figure 4), it can be seen that the outliers of the turning angle existing in the entire point cloud data (approximately 100° < θ ≤ 180°) and the existence range of the noise data (110° < θ ≤ 180°) are close to each other (Figure 4A,B). The *S. litura* point cloud data are without the data of the turning angle of the outlier (Figure 4B), and it is shown that the proportion of point cloud data of the corresponding turning angle exists at a high frequency in the noisy point cloud data (Figure 4C). However, in contrast to the turning angle, the speed was not useful for classifying the *S. litura* point cloud data and the noisy point cloud data (Figure 4).

### 3.3. Flight Speed Based on 3D Point Cloud Time Series

We obtained information on the free-flight speed of *S. litura* based on centroids of the measurement point cloud only from the *S. litura* point cloud data. This speed information was compared with the flight speed data from previous studies using flight mills. The mean ± SD of the flight speed of free-flying *S. litura* was 1.81 ± 0.68 m/s, and the median was 1.75 m/s, while the maximum flight speed was 0.88 ± 0.33 m/s (mean ± SD, non-laying adult females 3 days after eclosion) in a previous study [22] in which the mean value and standard deviation were approximately half the results of this study (Figures S1–S3, Video S1).

## 4. Discussion

In this study, we obtained noise-removed flight trajectories of free-flying *S. litura* by recording videos using infrared light and stereo cameras, the results of which were then processed using the 3D point cloud time series pipeline. Noise data with an OBV of 0 existed only around 0.9 m from the stereo camera. The distance of 0.9 m is analogous to the minimum effective range of the stereo camera. That is, it is difficult to accurately measure the *S. litura* point clouds near the effective range limit of the stereo camera. In this study, candidate data with large turning angles (110° < θ ≤ 180°) were noise data that were false positives in the previous pipeline. The turning angle is critical information as an effective criterion for removing these false positive data. Adding this information to the 3D tracking pipeline for free-flying *S. litura* can improve noise removal in the pipeline. There are two types of noisy point cloud data: noise-only point clouds and a mixture of the *S. litura* flight trajectory and noise point clouds. Therefore, flight trajectory data containing both *S. litura* and noise data must be partially adopted for the practical application of pest control by laser zapping. For this reason, a new pipeline may be required to deal with noisy 3D point clouds by early detection and substitution to a realistic position in the trajectory. The higher resolution and frame rate of the stereo camera imaging may capture free-flying *S. litura* in more detail. The accuracy of this measurement system may be improved by testing and further calibrating the denoising method based on *S. litura* tethered to flight mills or other models with a speed control motor within the 3D point cloud measurement system developed in this study.

Flight speed is an important factor for designing an automatic tracking and laser-based control system against pest insects. The flight speed data in this study were approximately twice as large as the speed data measured using a flight mill in a previous study [22] (flight mill in a previous study: 0.88 ± 0.33 m/s, 25 °C; free flight in this study: 1.81 ± 0.68 m/s, 24 °C, mean ± SD). In another study by Tu et al. (2010) that used a flight mill, the average flight speed was 1.14 ± 0.075 m/s (mean ± SE, unmated female 3 days after eclosion, 24 °C) [23]. The speed was faster than the speed in the study by Noda and Kamino (1988) [22], but the free-flight speed in this study was approximately 1.6 times larger than the speed in the study by Tu et al., which was obtained using a flight mill (this study: 1.81 ± 0.68 m/s, mean ± SD; the study by Tu et al.: 1.14 ± 0.075 m/s, mean ± SE). One reason for this result is the absence of behavioral limitations in this study compared to tethering on flight mills in previous studies. The stress from the catch and release by humans might have increased the flight speed as an escape behavior. In addition, UV light may have stimulated the flying behavior of moths. To accurately measure free-flying moths, it is necessary to develop an autonomous system for measuring the natural flight trajectory by minimizing human and UV light stimuli in a closed system, and in an ideal case, it should be measured under open, natural conditions. The method of this study enabled measurements to be obtained under dark or low-light conditions, and it became possible to measure the complex 3D flight trajectories and speed, including sudden changes in the direction and deceleration of nocturnal flying insects, such as *S. litura*, for which detailed analyses of flight have been difficult. To the best of our knowledge, there is no other method for the detailed measurement of 3D flight trajectories under low-light and free-flying conditions at a high resolution. These changes cannot be observed using a measurement method such as flight mills. In previous studies, insect flight speed has often been measured using flight mills or tethering with a string [22,23,24,25]. Recently, a system for measuring the 1D flight speed of insects by combining the Scheimpflug principle and LiDAR was proposed [26]. Despite the importance of effective pest control using laser zapping, data on the flight ability of *S. litura* remain scarce. Therefore, further investigations are required to accumulate more data, considering the various factors affecting the behavior of *S. litura*.

Previous studies on flying insects other than moths have been conducted. For instance, the flight behavior of dragonflies was observed using a single camera and a projected comb fringe [27]. The flight behavior of hoverflies was observed using two black-and-white cameras positioned above and one on the side [28]. In one study, the flight speed and distance of flying Hemiptera insects were estimated using a speedometer and information from flight mills [29]. However, this speedometer-based method is only suitable for almost linear movements and is vulnerable to electromagnetic noise, thereby limiting the applicability to such measurements.

Many efforts have been made to combine the objects in each camera and frame from the same individual to obtain 3D trajectories. Studies have matched the feature trajectories of each object using Relative Epipolar Motion (REM) [30], improved matching and tracking using an additional third camera [31,32], and a method to terminate tracking and link unterminated tracking in ambiguous situations [33]. Also, 3D trajectories of a large number of swarming animals were quickly obtained by combining paired trajectories with 2D images [34]. In contrast, our method can acquire faster and higher-resolution 3D trajectories, but it was only tested for the target species. The pipeline of this study could be improved and customized for other species by combining the techniques and knowledge of previous studies.

In our previous study [18], the laser-zapping system using the noise-removal pipeline could predict the positions of two frames (36 ms) ahead of the software simulation moths in real time. The hit rate was 70.1%, and the accuracy of the position prediction was 1.4 cm in the same simulation system. The accuracy of the position prediction can be improved by combining the information obtained in this study. To cope with noisy 3D point clouds in real-time settings, the coordinates, flight speed, and turning angle in the current frame may be used to improve the short-term prediction of free-flying *S. litura* flight trajectories. Considering the time lag between stereo camera imaging and laser zapping, a fast and accurate short-term prediction is indispensable for effective pest control. Furthermore, this measurement system can be applied to investigate the flight ability of other flying insects by adjusting the detection settings. However, it should be confirmed that this method can be applied to species with hovering and/or reverse flights. In the case of nocturnal flying insects, the observation of a more natural state of flight ability may be preferred compared to conventional measurement methods because visible light and tethering are not necessary. Further improvement of laser-based pest control will benefit from such information as the flight speed of flying pest insects under natural conditions.

This study presented a measurement system that allows for the observation of flight behavior under less restricted conditions. This method has the potential to provide new ecological information that has yet to be explored. Additionally, it can be used to study a wide range of flying species by enhancing and broadening the measurement system. For instance, a study that examined the influence of sidewind on bumblebee flight trajectories utilized a relatively small flight arena (48 × 48 cm) and verified that bumblebees could land accurately on the target even under the presence of sidewind [35]. It would also be possible to investigate the flight trajectories of a target species over larger areas and/or under low-light conditions using the methods proposed in this study. In the future, further development to enhance camera performance will allow for detailed information to be obtained over a broader range or with greater resolution than that in this study. This improvement can also facilitate studies related to the optimization of flight trajectories in flying animals.

## Figures and Tables

**Table 1:** Specification of stereo camera system (Karmin3 with SceneScan Pro, Nerian Vision Technologies, Stuttgart, Germany) used in recordings.

Camera	Karmin3
Processor	SceneScan Pro
Distance between cameras	10 cm
Resolution	1024 × 768 pixels
Horizontal angle	47.2°
Vertical angle	36.3°
Spatial resolution	1.6 mm (at 2 m distance)
Frame rate	55 fps

## Data Availability

The datasets and programs used in the current study are not publicly accessible due to confidentiality agreements.

## Supplementary Materials

The following supporting information can be downloaded at: https://www.mdpi.com/article/10.3390/insects15060373/s1, Figure S1: Graphs of turning angles from 10 *S. litura* trajectories; Figure S2: Graphs of speeds from 10 *S. litura* trajectories; Figure S3: Three-dimensional graphs of point cloud coordinates from 10 S. litura trajectories; Video S1: These videos visualized from time-series 3D point cloud data.
