# Supplementary material for: Measuring the Flight Trajectory of a Free-Flying Moth on the Basis of Noise-Reduced 3D Point Cloud Time Series Data
Source: Insects. 2024 May 21;15(6):373. doi: 10.3390/insects15060373 (PMC11203875; doi:10.3390/insects15060373)
Supplement: Supplementary file 1 [file insects-15-00373-s001.zip › Figures S1-S3.pdf]

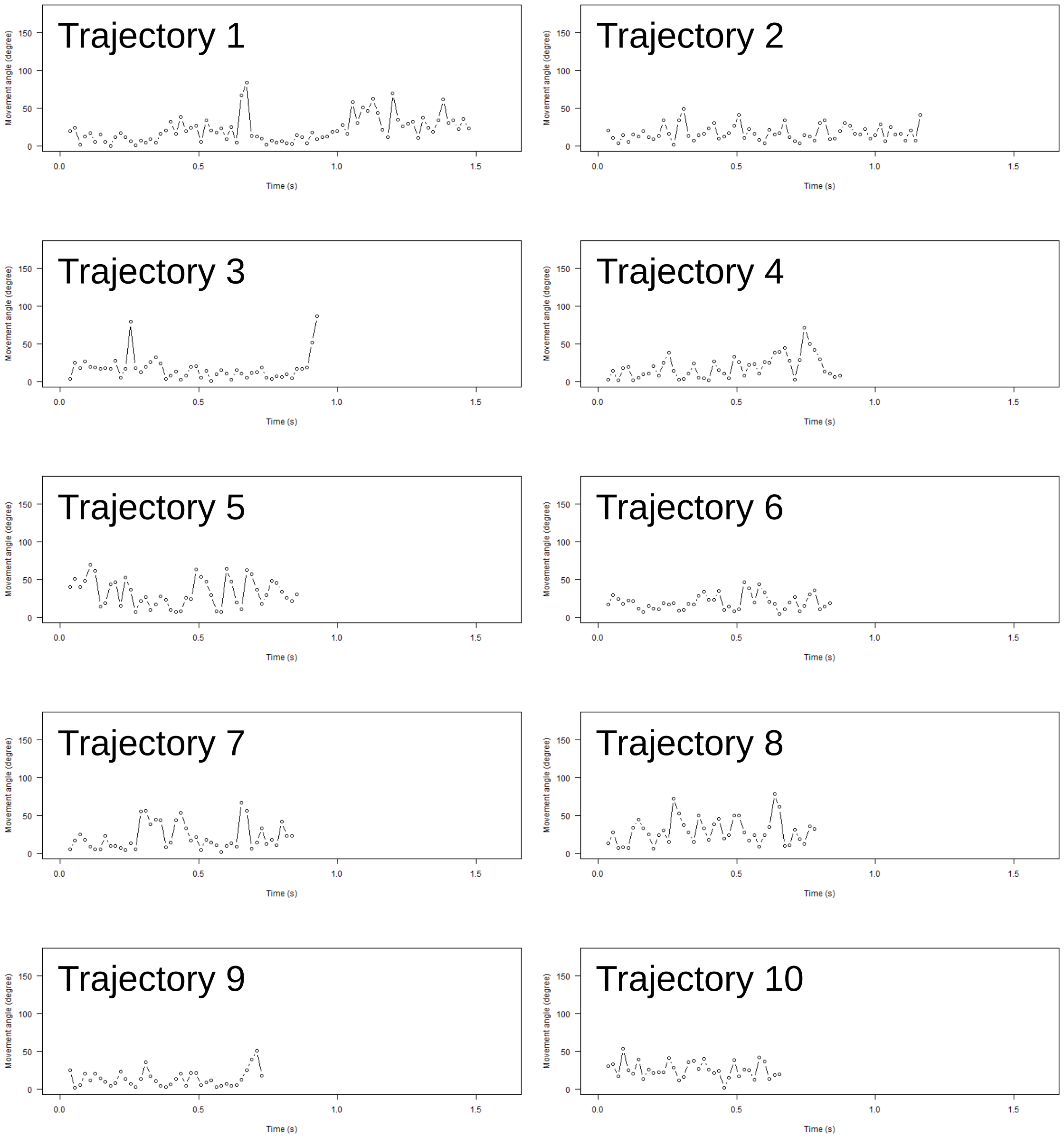

Figure S1. Graphs of turning angles from 10 *S. litura* trajectories, which are listed according to the length of time steps.

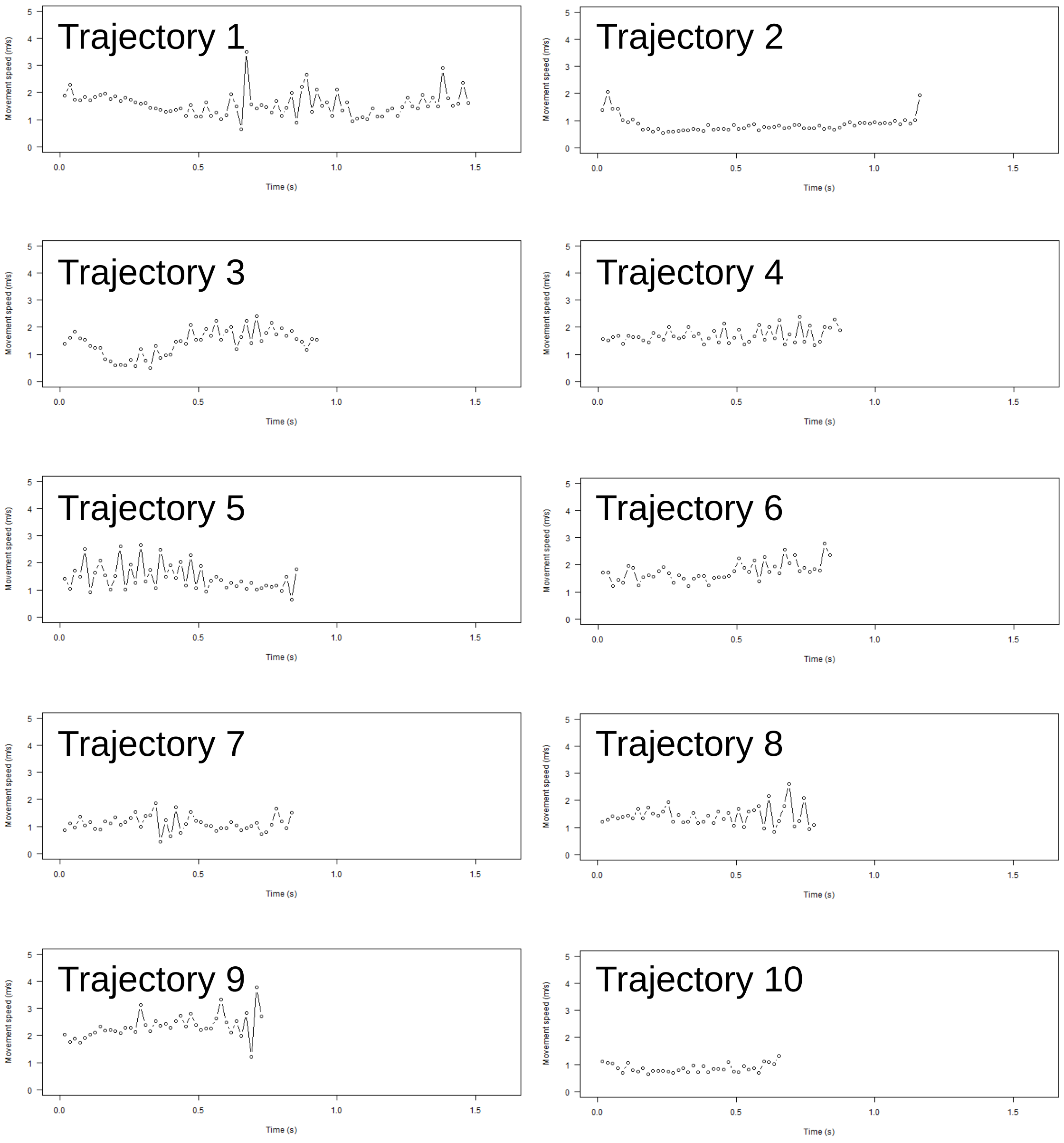

Figure S2. Graphs of speeds from 10 *S. litura* trajectories, which are listed according to the length of time steps.

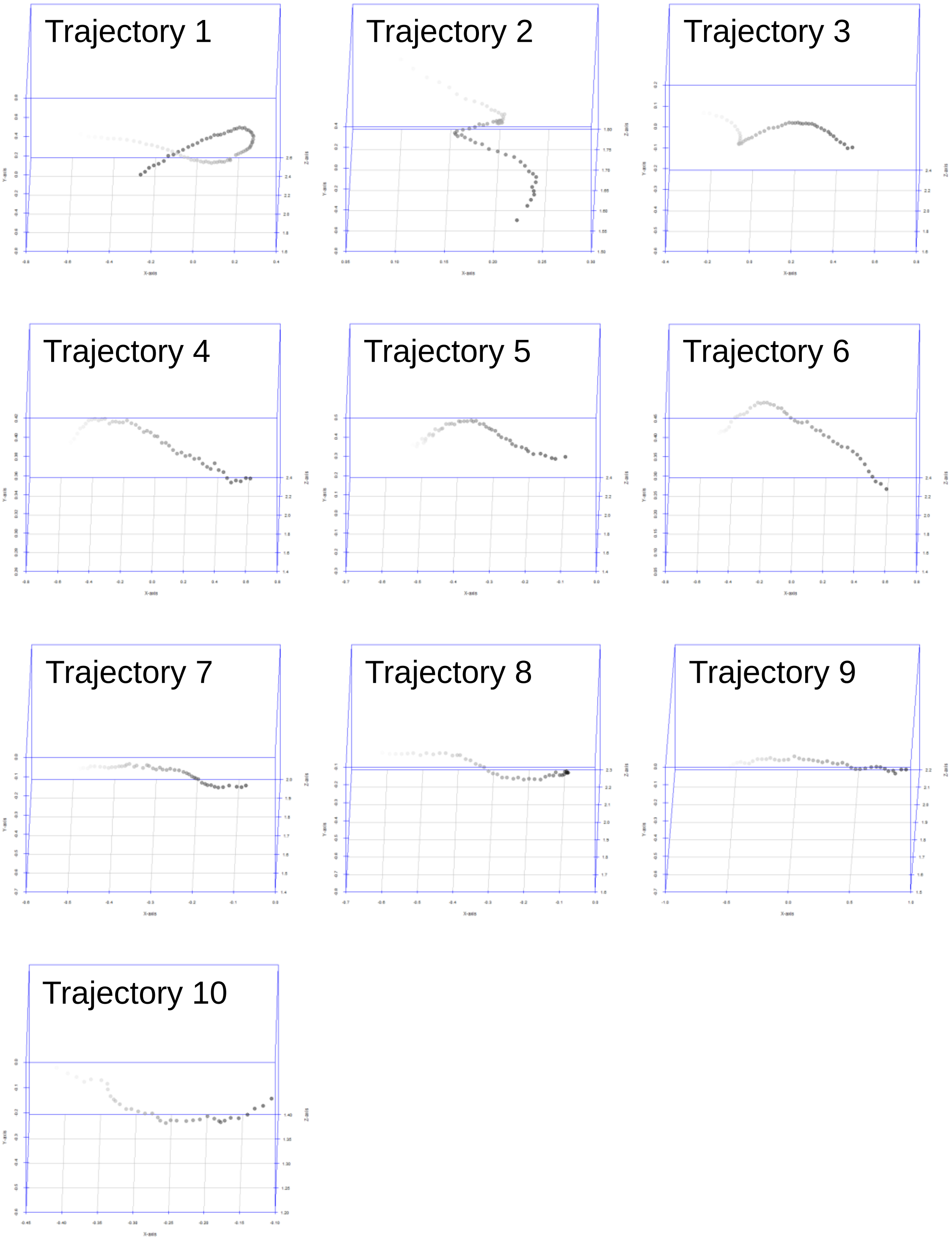

Figure S3. Three-dimensional graphs of point cloud coordinates from 10 *S. litura* trajectories, which are listed according to the length of time steps. Dark gray points indicate earlier time steps and light gray points indicate later time steps.
