# Supplementary material for: Measuring the Flight Trajectory of a Free-Flying Moth on the Basis of Noise-Reduced 3D Point Cloud Time Series Data
Source: Insects. 2024 May 21;15(6):373. doi: 10.3390/insects15060373 (PMC11203875; doi:10.3390/insects15060373)
Supplement: Supplementary file 1 [file insects-15-00373-s001.zip › Videos S1.zip!/Video_S1.pdf]

Video S1. These videos were visualized from time-series 3D point cloud data using ParaView. Letters A through D correspond to those in Figure 3. These are 1/5 times the speed. They were recorded at 55 fps and encoded at 11 fps.
